# Potential of *Pycnoporus sanguineus* (NUOL-PPK.02) for Bioremediation of Chlorpyrifos and Azoxystrobin in an Autoclaved Soil-Based Biomixture: A Pot Incubation Study

**DOI:** 10.3390/toxics14100839

**Published:** 2026-09-22

**Authors:** Nuttaya Xasydavong, Phoutthasone Sibounnavong, Noppol Arunrat, Jarupong Prasopsuk, Chuleemas Boonthai Iwai

**Affiliations:** 1 Department of Soil Science and Environment, Faculty of Agriculture, Khon Kaen University, Khon Kaen 40002, Thailand; nuttaya.x@kkumail.com; 2 Department of Soil Science, Crop Science and Plant Protection, Faculty of Agriculture, National University of Laos, Vientiane 7322, Laos; p.sibounnavong@nuol.edu.la; 3 Faculty of Environment and Resource Studies, Mahidol University, Nakhon Pathom 73170, Thailand; noppol.aru@mahidol.ac.th; 4 Office of Agricultural Research and Development Region 3, Department of Agriculture, Ministry of Agriculture and Cooperatives, Khon Kaen 40000, Thailand; 5 Integrated Land and Water Resource Management Research and Development Center in Northeast Thailand, Khon Kaen University, Khon Kaen 40002, Thailand

**Keywords:** soil-based biomixture, biobed, fungal bioremediation, white-rot fungus, pesticide contamination, chlorpyrifos, azoxystrobin, strobilurin, *Pycnoporus sanguineus*

## Abstract

Chlorpyrifos and azoxystrobin are among the most heavily used pesticides in banana production in many parts of the world, including the Lao People’s Democratic Republic (Lao PDR) and Thailand. In these systems, soils may receive an organophosphate insecticide and a strobilurin fungicide together, often alongside copper-based products. This study evaluated the bioremediation potential of *Pycnoporus sanguineus* (NUOL-PPK.02), a white-rot fungus isolated from decaying wood in Lao PDR. A pot incubation experiment was carried out under controlled conditions in an autoclaved soil-based biomixture (soil, sawdust, rice bran and calcium carbonate) using a completely randomised design with three replicates. Treatments comprised an uncontaminated biomixture control, an uncontaminated biomixture inoculated with *P. sanguineus*, and three application rates of each pesticide with and without fungal inoculation, with the non-inoculated pesticide treatments serving as controls for abiotic dissipation. Chlorpyrifos was applied at 16.3, 24.5 and 32.7 mg a.i. kg^−1^ dry biomixture and azoxystrobin at 10.2, 15.3 and 20.4 mg a.i. kg^−1^; measured initial concentrations 24 h after application were 8.39–10.58 and 4.73–5.54 mg kg^−1^, respectively. Residues were quantified by liquid chromatography–tandem mass spectrometry. After 60 days of incubation, the inoculated biomixture had lost 32%, 23% and 22% of chlorpyrifos and 76%, 60% and 53% of azoxystrobin, compared with 28%, 20% and 18% and 27%, 28% and 36% in the corresponding non-inoculated controls; differences were significant for azoxystrobin (Tukey’s HSD, *p* ≤ 0.05) but not consistently so for chlorpyrifos. The fungus tolerated both pesticides, although mycelial growth was inhibited by up to 51% (chlorpyrifos) and 60% (azoxystrobin), while available phosphorus, cation exchange capacity and organic matter content increased in the inoculated biomixture. These results indicate that *P. sanguineus* (NUOL-PPK.02) can accelerate the degradation of azoxystrobin under the conditions tested, while contributing little to the dissipation of chlorpyrifos, and that fungal inoculation concurrently improves biomixture chemical properties.

## 1. Introduction

Soil is crucial in agricultural productivity and sustainability, serving as the foundation for global food security. However, modern agricultural practices heavily rely on agrochemicals, particularly pesticides, to protect crops from unwanted plants, insects, and fungi. Since the first insecticides were applied in 1892, chemical pesticides have expanded dramatically, with over 80,000 chemicals now available for pest control and approximately 80–90% of applied chemical inputs used for this purpose [1]. While these chemicals are essential for maintaining crop yields, their persistence and toxicity in soil raise serious environmental concerns, including decreased biodiversity, chemical residue accumulation, and the uptake of harmful compounds throughout ecosystems.

The global banana industry exemplifies both modern agriculture’s economic importance and environmental challenges. Banana production has shown steady growth since 2010, reaching nearly 125 million tons by 2021, making it one of the world’s most important fruit crops and the most traded fruit globally in terms of export value [2]. Laos PDR and Thailand are major banana-producing countries in Southeast Asia, with the Cavendish variety comprising approximately 45% of global production and expanding into mainland China markets [3]. In Laos, banana plantations cover an estimated 19,811 hectares across 12 provinces, producing approximately 812,177 tons annually [4].

However, banana cultivation in the region faces severe threats from diseases and pests, necessitating intensive pesticide use. Fusarium wilt, caused by the soil-borne fungus *Fusarium oxysporum* f. sp. *cubense*, has emerged as the primary cause of yellow leaf symptoms in banana plants [5]. This devastating pathogen was first detected in northern Laos in 2016 [6]. It caused a significant outbreak in Chiang Rai in 2022, raising concerns for the global banana industry. The disease has caused substantial losses in Cavendish banana plantations across Malaysia, South China, and Australia, particularly in regions bordering Laos [7].

Increasing reliance on pesticides is particularly evident in developing Southeast Asian countries [8]. According to the World Health Organization, developing nations account for 20% of global pesticide use, with Cambodia importing 61% more pesticides annually, Laos at 55%, and Vietnam at 10% [9]. The Food and Agriculture Organization reported that global pesticide use reached 3.70 million tons of active ingredients in 2022, representing a 4% increase from 2021 and a doubling since 1990 [10]. In Laos, agricultural pesticide use totalled 891.82 tons in 2022, including 51.82 tons of fungicides and bactericides and 5.65 tons of organophosphate insecticides. Thailand’s pesticide consumption was significantly higher at 5806.02 tons, with 3223.63 tons of organophosphate insecticides and 1898.91 tons of fungicides [10].

Among the most commonly used pesticides in banana plantations are chlorpyrifos and azoxystrobin. Chlorpyrifos is a broad-spectrum organophosphorus insecticide applied to grain, field, fruit, and vegetable crops, lawns, and ornamental plants. It is available in various formulations including granular, emulsifiable, dust, wettable, and powder forms [11,12]. Chlorpyrifos exhibits moderate persistence in soils, with half-lives ranging from two weeks to over a year depending on soil type, climate, and other environmental factors, typically between 60 and 120 days. The compound can be degraded by soil microorganisms, UV light, and chemical hydrolysis [13,14]. Azoxystrobin is a synthetic methoxy acrylate fungicide widely used globally to treat fungal diseases in fruits, vegetables, peanuts, and other crops [15,16,17]. The two compounds differ markedly in their behaviour in soil. According to the Pesticide Properties Database [18], the typical aerobic soil DT_50_ of azoxystrobin is 78 days (laboratory 84.5 days at 20 °C; field 180.7 days), with reported ranges of 35.2–248 days in laboratory studies and 120.9–261.9 days in the field, so that it is classed as moderately persistent to persistent. Chlorpyrifos is reported with a typical dark-aerobic soil DT_50_ of 386 days and a field DT_50_ of 27.6 days, with the laboratory range spanning 19.8 to more than 1000 days across eight soils [19]; the discrepancy between laboratory and field values reflects the importance of volatilisation and photolysis in the field rather than rapid biodegradation, and chlorpyrifos is not readily biodegradable. The compounds also differ by an order of magnitude in their affinity for organic matter, with K_oc_ values of about 5509 mL g^−1^ for chlorpyrifos against 589 mL g^−1^ for azoxystrobin [18,19], a contrast that proves central to the results reported here. The regulatory status of chlorpyrifos nevertheless differs markedly between regions. The European Union did not renew the approval of chlorpyrifos, and Member States were required to withdraw all authorisations for plant protection products containing it by February 2020 [20]. In contrast, the compound remains registered and widely applied in many tropical banana-producing countries, including those of Southeast Asia [10], so that soils in these regions continue to receive chlorpyrifos inputs long after its phase-out elsewhere. Remediation approaches suited to tropical agricultural soils therefore remain a pressing need.

Reported residues of both compounds in agricultural soils are substantial. Chlorpyrifos has been detected in cultivated soils at concentrations from below 0.01 to more than 10 mg kg^−1^, and azoxystrobin at up to several mg kg^−1^ in intensively treated systems, and both compounds affect non-target soil organisms at concentrations within this range. The chronic no-observed-effect concentration for earthworm reproduction is 0.075 mg kg^−1^ dry soil for chlorpyrifos and 3.0 mg kg^−1^ for azoxystrobin, with acute 14-day LC_50_ values of 129 and 283 mg kg^−1^, respectively, for *Eisenia fetida*, and the chronic collembolan NOEC for chlorpyrifos is 0.012 mg kg^−1^ [18,19]. Remediation of soils receiving repeated applications is therefore warranted. Bioremediation is a promising, eco-friendly technology that utilises fungi, bacteria, and plants to remediate contaminated soil [21]. This innovative approach is particularly attractive because it works harmoniously with natural ecosystems [22]. Fungal bioremediation employs mycelium to break down contaminants in soil environments. Fungi are particularly effective because they produce enzymes capable of degrading numerous contaminants while using these compounds as food sources [23,24]. Chlorpyrifos, an organophosphorothioate, is degraded principally through hydrolysis of the phosphorothioate ester, yielding 3,5,6-trichloro-2-pyridinol (TCP); azoxystrobin, a strobilurin, is attacked at the methoxyacrylate side chain. White-rot fungi mediate both classes of reaction through non-specific extracellular oxidative enzymes—laccase, lignin peroxidase and manganese peroxidase—supplemented by intracellular cytochrome P450 monooxygenases, and this non-specificity is the biochemical basis of their broad substrate range and of their tolerance to structurally diverse xenobiotics [25,26]. These organisms can tolerate wide ranges of environmental conditions and soil pollutants, and their metabolic capabilities allow them to break down complex compounds, earning them recognition as nature’s ultimate decomposers of stubborn organic materials [27]. The effectiveness of fungal bioremediation depends on various extracellular compounds and metabolites that facilitate transformative interactions between fungi and contaminants [28,29]. Fungi can absorb heavy metals through ionic interactions and complex formation between metal ions and functional groups on cell surfaces [30]. Additionally, fungi play crucial roles in stabilising soil organic matter, immobilising and retaining nutrients, and decomposing residues, with their activities significantly influenced by the physicochemical and ecological interactions within soil systems [31].

Pesticide degradation by introduced fungi depends strongly on the matrix in which it takes place. Much of the work on fungal bioaugmentation has accordingly been carried out in biomixtures rather than in natural soil. Biomixtures are the biologically active core of biobeds and related on-farm biopurification systems. These systems were developed in Sweden in the 1990s to retain and degrade pesticides released from spills and from the filling of spraying equipment, and modified designs in other countries also treat washing water [32]. The classical formulation combines a lignocellulosic material such as straw, a humified material such as peat or compost, and soil, typically in a volumetric ratio of 2:1:1 [32,33]. The lignocellulosic fraction supplies carbon and sustains microbial activity. The humified fraction increases sorption capacity and regulates moisture content and pH. The soil contributes sorption sites and, in non-sterile systems, a degrading microflora [32,33]. Locally available agricultural and forestry residues have been used in place of straw and peat in many regions [33]. A new formulation is commonly evaluated first in a biomixture assay, that is, a laboratory or pot incubation of pesticide-spiked biomixture under controlled conditions, before it is applied in a biobed [33]. Lignin-rich materials also support the ligninolytic enzyme system of white-rot fungi, in which laccases and peroxidases oxidise a broad range of organic pollutants [34]. Bioaugmentation of biomixtures with these fungi has therefore been investigated as a way of accelerating pesticide removal, with outcomes that depend on the fungal strain, the pesticide and the composition of the biomixture [33]. A soil-based biomixture amended with sawdust and rice bran is thus an appropriate first matrix in which to test the degradative capacity of a newly isolated white-rot fungus. Results obtained in such a matrix, however, cannot be extrapolated directly to natural agricultural soils.

*Pycnoporus sanguineus* (Basidiomycota, Polyporales) is a pantropical white-rot fungus that colonises dead hardwood in tropical and subtropical forests and is readily cultivated on lignocellulosic substrates and on diverse laboratory media [35,36]. It is distinguished by its bright orange to vermilion basidiocarps and by high constitutive laccase production. Species of *Pycnoporus* produce cellulases, hemicellulases, pectinases and laccases, all contributing to their pollutant degradation capabilities [36], and *P. sanguineus* and related white-rot fungi have been reported to transform a range of xenobiotics, including organophosphate and strobilurin pesticides, polycyclic aromatic hydrocarbons and synthetic dyes [36,37]. This oxidative enzyme activity toward structurally diverse organic pollutants is directly relevant to banana-plantation soils, which receive an organophosphate insecticide and a strobilurin fungicide of contrasting degradation chemistry, frequently alongside copper-based products, so that any candidate remediation agent must act on more than one class of compound. Therefore, this study aims to evaluate the bioremediation potential of *P. sanguineus* (NUOL-PPK.02), a strain isolated from decaying wood in Lao PDR, for degrading chlorpyrifos and azoxystrobin in an autoclaved soil-based biomixture under controlled pot incubation conditions, as a first step towards its use in biobed-type treatment systems and, subsequently, in agricultural soils.

## 2. Materials and Methods

### 2.1. Pure Culture Source

The strain *P. sanguineus* (NUOL-PPK.02) was provided by the Plant Protection Unit, Department of Soil Science, Crop Science and Plant Protection, Faculty of Agriculture, National University of Laos. It is not an ATCC or other culture-collection accession. The strain was isolated in 2019 from basidiocarps growing on decaying hardwood collected in Phou Khao Khouay National Park, Bolikhamxay Province, Lao PDR. It was identified morphologically: colony growth pattern, colour, texture and margin characteristics were examined on agar medium under a stereomicroscope, and the morphology and arrangement of hyphae and spores were examined under a compound microscope on cultures grown on potato dextrose agar (PDA) for 14–21 days; the characters observed were compared with published taxonomic descriptions and identification keys. Confirmation by internal transcribed spacer (ITS) rDNA sequencing is in progress, so the species assignment reported here rests on morphological characters alone, which is noted as a limitation in Section 4. For use in the experiment, the culture was aseptically transferred onto sterilised PDA and incubated at 30 °C for 14 days. To prepare grain spawn, whole sorghum grains were boiled in water until approximately 30% cooked, judged visually from the slightly opaque white appearance of the endosperm, then cooled and packed into heat-resistant bags at 25 g of grain per bag and autoclaved at 121 °C for 4 h. After cooling to room temperature, each bag was inoculated with one agar plug (5 mm diameter) cut from the actively growing edge of a 14-day-old PDA culture and incubated under sterile conditions at 25 °C for 14 days, until the grain was fully colonised by dense, dark-orange mycelium. Fully colonised spawn was used as the inoculum.

### 2.2. Soil Sample Source

The soil used in this study belongs to the Korat soil series, classified as a fine-loamy, siliceous, isohyperthermic Typic Paleustult in USDA Soil Taxonomy and corresponding to a Haplic Acrisol (Arenic, Khon Kaen, Thailand) in the World Reference Base for Soil Resources. It was collected from the 1–10 cm layer of an organic agricultural field with no history of pesticide application at the nursery of the Department of Soil Science and Environment, Faculty of Agriculture, Khon Kaen University, Khon Kaen, Thailand. The Korat series is a sandy, strongly weathered upland soil of low inherent fertility, typically with less than 1% organic matter, low cation exchange capacity and acidic reaction; the properties of the batch used here are given in Table 1. Samples were air-dried, sieved through a 90-mesh sieve to remove plant debris and stones, and stored in sealed polyethylene bags at room temperature in the dark for 7 days until use.

### 2.3. Experimental Set-Up and Contaminant Composition

The experiment followed a modified method based on Adenipekun and Fasidi [37] and was arranged as a completely randomised design with 14 treatments and three replicate containers per treatment (42 containers in total). Commercial formulations were used: chlorpyrifos 40% *w*/*v* emulsifiable concentrate (TIGER; 400 g a.i. L^−1^) and azoxystrobin 25% *w*/*v* suspension concentrate (MISTA; 250 g a.i. L^−1^). For each application rate, 100, 150 or 200 µL of formulated product was pipetted into 200 mL of distilled water and applied evenly to the biomixture in one container, which was then mixed thoroughly. The nominal application rates were 16.3, 24.5 and 32.7 mg a.i. kg^−1^ dry biomixture for chlorpyrifos and 10.2, 15.3 and 20.4 mg a.i. kg^−1^ for azoxystrobin; the derivation is given in Supplementary Table S1. For reference, the manufacturers’ recommended field rates are 60 mL product per 20 L water for chlorpyrifos (1200 mg a.i. L^−1^ spray solution) and 15 mL per 20 L for azoxystrobin (187.5 mg a.i. L^−1^); the rates used here correspond to spray solutions of 200–400 and 125–250 mg a.i. L^−1^, respectively, and were selected to represent residue concentrations that accumulate in repeatedly treated plantation soils rather than a single field application. Two uncontaminated controls were included: untreated biomixture and biomixture inoculated with grain spawn of *P. sanguineus* (NUOL-PPK.02). Critically, each pesticide application rate was represented by a pair of treatments—one inoculated with *P. sanguineus* and one non-inoculated. So that fungal degradation could be separated from abiotic dissipation (hydrolysis, sorption and any residual chemical loss) in the same matrix. Treatments are set out in full in Table 2. The two pesticides were applied to separate containers and were never combined in the same container.

The biomixture was prepared as follows: Sieved soil (2000 g), sawdust (200 g), rice bran (200 g) and calcium carbonate (50 g) were mixed thoroughly, giving 2450 g dry weight per container. Sawdust was included as a lignocellulosic carbon source to support the ligninolytic enzyme system of the white-rot fungus, rice bran as a nitrogen and micronutrient source, and calcium carbonate to buffer the acidic Korat soil to a pH suitable for fungal colonisation; this formulation follows standard practice for cultivating white-rot basidiomycetes on lignocellulosic substrates. The resulting matrix is accordingly a soil-based biomixture of the type used in biobed and bioreactor systems, not a natural soil, and the results should be interpreted as demonstrating the capacity of the fungus under controlled bioreactor-like conditions rather than as predicting behaviour in undisturbed field soil. The mixture was weighed into containers of 3 L capacity, filled to approximately three-quarters of their volume, sealed in bags and sterilised in an autoclave at 121 °C for 4 h. Sterilisation substantially alters the physical, chemical and biological properties of the matrix, including the elimination of the indigenous microbial community and the release of labile carbon and nitrogen; this is acknowledged as a limitation and means that the non-inoculated controls represent abiotic dissipation in a sterile matrix rather than natural attenuation in a living soil. After cooling, the pesticide solutions were applied as described above and the biomixture mixed. Inoculated containers then received 25 g of fully colonised grain spawn of *P. sanguineus* (NUOL-PPK.02) in a laminar flow cabinet, 16 h after pesticide application. Containers were closed with a lid, arranged on shelves in a temperature-controlled incubation room at 30 ± 2 °C for 60 days, and opened only for sampling. Water was supplied by the 200 mL of application solution added to each container; uncontaminated control containers received 200 mL of distilled water. The moisture content of the biomixture, determined gravimetrically on a wet-weight basis at the start of the incubation, was 30.4–33.2% in the chlorpyrifos experiment and 30.4–37.1% in the azoxystrobin experiment (Supplementary Tables S11 and S12). Moisture was not adjusted during the incubation: no water was added after application, and the containers remained closed except at sampling. Moisture content after 30 days was 11.3–27.2% and is discussed in Section 3.3. The experiment was carried out at the Vermitechnology Research Centre for Sustainable Agriculture and Environment, Faculty of Agriculture, Khon Kaen University, Thailand.

### 2.4. Sample Collection and Analytical Methods

Biomixture was sampled at 0, 7, 14, 30 and 60 days. The day 0 sample was taken 24 h after pesticide application. Inoculation of the inoculated containers was carried out 16 h after pesticide application, so the day 0 sample was withdrawn 8 h after the spawn was added; because mycelial establishment had not yet begun at that point, the day 0 value is treated as a common measured initial concentration for the inoculated and non-inoculated containers of the same application rate. At each sampling, the full contents of each container were mixed and a 50 g composite sample withdrawn. Initial properties of the biomixture components were determined before the experiment was established (Table 3), and the physicochemical properties of the biomixture were determined at the start and after 30 days of incubation.

Biomixture chemical and physical properties were determined as follows: pH was measured in a 1:2 soil-to-water suspension [38] and electrical conductivity in the same extract [39]. Cation exchange capacity was determined by ammonium acetate saturation (1 N NH_4_OAc, pH 7.0). Total nitrogen was determined by Kjeldahl digestion, distillation and titration; available phosphorus by Bray and Kurtz No. 2 extraction with colourimetric determination; exchangeable potassium by 1 N NH_4_OAc extraction followed by atomic absorption spectrophotometry; and organic matter and organic carbon by the Walkley–Black wet oxidation method, with the C:N ratio calculated from organic carbon and total nitrogen. Moisture content was determined gravimetrically by oven-drying at 105 °C to constant weight and is expressed on a wet-weight basis.

Pesticide residues were determined at the Office of Agricultural Research and Development Region 3, Department of Agriculture, Khon Kaen, Thailand. The laboratory applies this multi-residue procedure routinely to agricultural produce from the region [40]. Samples were extracted according to the QuEChERS procedure of BS EN 15662:2018 [41] using anhydrous magnesium sulfate (4.0 ± 0.2 g), sodium chloride (1.00 ± 0.05 g), disodium hydrogencitrate sesquihydrate (0.50 ± 0.03 g) and trisodium citrate dihydrate (1.00 ± 0.05 g), and cleaned up by dispersive solid-phase extraction with anhydrous magnesium sulfate (600 ± 30 mg), primary secondary amine (100 ± 5 mg) and graphitised carbon black (10 ± 1 mg). Analysis was performed on a Thermo Scientific Ultimate 3000 liquid chromatograph (Thermo Fisher Scientific, Inc., Dornierstraße 4 D-82110 Germering, Germany) coupled to a Bruker Compact ESI-Q-TOF mass spectrometer (Bruker Daltonics GmbH & Co. KG, Fahrenheitstraße 4, 28359 Bremen, Germany) using a Thermo Scientific Acclaim RSLC 120 C18 column (Thermo Fisher Scientific Baltics UAB V. A. Graičiūno g. 8, LT-02241 Vilnius, Lithuania) (2.1 × 100 mm, 2.2 µm, 120 Å) at 30 °C with an injection volume of 5 µL. Mobile phase A was 10% methanol and mobile phase B was methanol, both containing 5 mM ammonium formate and 0.01% formic acid; a multi-step gradient was applied over a 20 min cycle at 0.20–0.48 mL min^−1^ (Supplementary Table S2). The instrument was operated with positive electrospray ionisation (capillary 4500 V, end-plate offset 500 V, drying gas 8.0 L min^−1^ at 180 °C, nebuliser 2.0 bar), acquiring spectra in broadband collision-induced dissociation mode over *m*/*z* 30–1000 with a 3.0 s cycle time. The quadrupole was operated at an ion energy of 4 eV with a low-mass cut-off of 50 *m*/*z* and an isolation width of ±0.5 *m*/*z*; the collision cell was operated at a collision energy of 4 eV with a collision RF of 200–700 Vpp and a transfer time of 20–70 µs. Quantification was based on extracted ion chromatograms of the quantifier product ion, with identity confirmed by a qualifier product ion within a mass accuracy of ±5 ppm (Supplementary Table S4). Matrix-matched calibration standards were prepared in blank biomixture extract at five concentrations: 0.005, 0.01, 0.05, 0.1 and 0.2 mg L^−1^. Extraction used 10 g of biomixture in 10 mL of acetonitrile, so that 1 mg L^−1^ in the extract corresponds to 1 mg kg^−1^ in the biomixture and the reported limits of detection and quantification apply equally on either basis. Extracts whose response exceeded the highest calibration point were diluted and re-injected, so that every value reported here falls within the calibrated interval; the dilution factors applied were not recorded. Linearity (r^2^ ≥ 0.995), limits of detection (0.001 mg kg^−1^) and quantification (0.01 mg kg^−1^), recovery (90.9% for azoxystrobin, 114.2% for chlorpyrifos) and precision (RSD < 6%) are summarised in Supplementary Table S5. Recovery was assessed by spiking blank biomixture at 0.1 mg kg^−1^ in triplicate. All residue concentrations are expressed on a dry-weight basis.

Mycelial extension was measured as the vertical distance from the biomixture surface, taken as the reference point, to the lowest visible point of mycelial growth against the container wall, recorded to the nearest 0.1 cm with a ruler at 7, 14 and 30 days; the procedure is illustrated in Supplementary Figure S1. One measurement was made per container at each sampling time, and the value reported for a treatment is the mean of its three replicate containers. These measurements, together with the visible mycelium documented photographically at the end of the incubation, were the basis on which colonisation of the biomixture was confirmed; no re-isolation on agar or measurement of fungal biomass was carried out, and this is acknowledged as a limitation. Removal of pesticide and inhibition of mycelial growth were calculated as follows: removal (%) = [(C_0_ − C_t_)/C_0_] × 100, where C_0_ is the measured concentration at day 0 and C_t_ the concentration at time t, applied to inoculated and non-inoculated containers alike; because this total removal includes abiotic losses, the removal attributable to the fungus was obtained by correcting for the non-inoculated control of the same application rate: fungal removal (percentage points) = [(C_ni,t_ − C_i,t_)/C_0_] × 100, where C_ni,t_ and C_i,t_ are the concentrations in the non-inoculated and inoculated containers at time t and C_0_ is the common measured day 0 concentration, so that fungal removal equals the difference between the total removals of the two treatments; inhibition of mycelial growth (%) = [(G^c^ − G_t_)/G^c^] × 100, where G^c^ is mycelial extension in the uncontaminated inoculated control and G_t_ that in the contaminated treatment at the same sampling time.

### 2.5. Statistical Analysis

Statistical analyses were performed using Statistix 10. Data were checked for normality (Shapiro–Wilk) and homogeneity of variance (Levene’s test) before analysis. Residue and biomixture property data were subjected to one-way analysis of variance (ANOVA) appropriate to the completely randomised design, and treatment means were separated using Tukey’s Honestly Significant Difference test at *p* ≤ 0.05. The two uncontaminated control treatments returned non-detectable pesticide residues at every sampling time and were therefore reported descriptively and excluded from the residue ANOVA, which compared the six pesticide treatments of each compound. Significance at *p* ≤ 0.001 is indicated by three asterisks (***)*;* means followed by the same letter within a column do not differ significantly. All treatments comprised three replicate containers, and results are expressed as the mean ± standard deviation. Pearson correlation coefficients were calculated between fungal growth, residue concentrations and biomixture properties.

## 3. Results and Discussion

### 3.1. Efficiency of Fungi Pycnoporus sanguineus (NUOL-PPK.02) in Biodegradation

#### 3.1.1. Dissipation of Chlorpyrifos in the Biomixture

Chlorpyrifos was applied at three nominal rates (16.3, 24.5 and 32.7 mg a.i. kg^−1^ dry biomixture), and residues were determined by LC-MS/MS (Figure 1). Measured concentrations 24 h after application (day 0) were 8.39 ± 0.32, 9.86 ± 0.32 and 10.58 ± 0.11 mg kg^−1^, corresponding to 51%, 40% and 32% of the nominal rates. This shortfall, and the fact that it increased with application rate, is addressed in Section 3.4; in brief, it reflects sorption to the highly organic biomixture during the 24 h before sampling, together with the analytical constraints discussed there. Because measured initial concentrations differed by only 1.26-fold across the three rates, the treatments are not interpreted as a dose–response series, and all dissipation parameters were calculated from measured rather than nominal concentrations. Residues declined in both inoculated and non-inoculated biomixture from day 7 onwards. After 30 days, concentrations in inoculated biomixture had fallen to 6.06, 8.05 and 8.76 mg kg^−1^ (reductions of 28%, 18% and 17%), compared with 6.37, 8.12 and 9.06 mg kg^−1^ in the non-inoculated controls (24%, 18% and 14%). After 60 days, inoculated biomixture contained 5.73, 7.60 and 8.27 mg kg^−1^ (reductions of 32%, 23% and 22%) against 6.05, 7.86 and 8.67 mg kg^−1^ without inoculation (28%, 20% and 18%). After correction for abiotic loss (Section 2.4), the removal attributable to *P. sanguineus* after 60 days was therefore only 3–4 percentage points at every rate and inoculated and non-inoculated biomixture did not differ significantly at any rate by day 60 (Tukey’s HSD, *p* ≤ 0.05); significant differences were recorded only at day 7, at the two higher rates. We therefore do not claim effective fungal degradation of chlorpyrifos under these conditions; the fungus survived and grew in chlorpyrifos-contaminated biomixture, but dissipation was dominated by abiotic processes.

The limited effect on chlorpyrifos contrasts with the marked effect on azoxystrobin reported below, and two explanations are consistent with the data. First, chlorpyrifos is strongly sorbed in organic-rich matrices (log K_oc_ ≈ 4.7), so that much of the applied compound is likely to have been unavailable to extracellular fungal enzymes. Second, the phosphorothioate ester of chlorpyrifos is less readily attacked by the laccase and peroxidase system of white-rot fungi than the methoxyacrylate side chain of azoxystrobin. Fungal removal of chlorpyrifos has been reported under other conditions: fungi isolated in northern Egypt tolerated chlorpyrifos at up to 2000 mg kg^−1^, although they removed only 24.16% of it under laboratory conditions [42], whereas Lakshmi et al. [40] reported removal of 85–92% after 30 days of incubation. The contrast between the present results and those of Lakshmi et al. [43] is most plausibly attributable to differences in matrix organic matter content and hence in bioavailability, and to differences in fungal species and inoculum density. Various microorganisms, particularly fungi, have been employed for the bioremediation of pesticide-contaminated environments [44,45,46]; the molecular structure and functional groups of a pesticide govern the specificity of degradation reactions across fungal species [47,48].

#### 3.1.2. Biodegradation of Azoxystrobin in the Biomixture

Azoxystrobin residues in the biomixture were determined by LC-MS/MS, as shown in Figure 2. Significant differences in residue concentrations were observed across concentration levels and incubation times. At the initial stage, azoxystrobin was applied at 10.2, 15.3 and 20.4 mg a.i. kg^−1^, and mean residue concentrations were 4.73 mg kg^−1^, 4.81 mg kg^−1^, and 5.54 mg kg^−1^, respectively. Azoxystrobin residues in inoculated biomixture declined from day 7 onwards and decreased further after 14 days of incubation. Over this period, *P. sanguineus* continued to grow and altered its morphology under the contaminated conditions, although whether it used azoxystrobin as a growth substrate was not determined. At 30 days, residues had decreased significantly to mean values of 2.13 mg kg^−1^, 2.26 mg kg^−1^ and 3.33 mg kg^−1^, respectively, corresponding to total reductions of 55%, 53% and 40%, compared with 24%, 22% and 22% in the non-inoculated controls; after correction for abiotic loss (Section 2.4), 31, 31 and 18 percentage points of this removal were attributable to *P. sanguineus*. After 60 days of incubation, residues had decreased further to mean values of 1.14 mg kg^−1^, 1.94 mg kg^−1^ and 2.63 mg kg^−1^, corresponding to total reductions of 76%, 60%, and 53%, respectively, before correction for abiotic loss.

In contrast to chlorpyrifos, *P. sanguineus* substantially accelerated the dissipation of azoxystrobin. After 60 days, removal in inoculated biomixture reached 76%, 60% and 53% at the three application rates, against 27%, 28% and 36% in the corresponding non-inoculated controls, and the differences were significant at every rate and every sampling time from day 7 onwards (Tukey’s HSD, *p* ≤ 0.05). After correction for abiotic loss using the non-inoculated controls (Section 2.4), the removal attributable to *P. sanguineus* was therefore 49, 32 and 17 percentage points at 10.2, 15.3 and 20.4 mg a.i. kg^−1^, respectively, an order of magnitude larger than for chlorpyrifos, and it is on azoxystrobin that the bioremediation potential of this strain rests. It should be noted that measured initial concentrations across the three application rates differed by only 1.17-fold (4.73, 4.81 and 5.54 mg kg^−1^), so the apparent decline in removal efficiency with increasing rate should be interpreted with caution and is not presented as a dose–response relationship. Fungicides are organic compounds, and many soil microorganisms have evolved the capacity to utilise them as carbon and energy substrates [49,50]. The methoxyacrylate side chain of azoxystrobin is susceptible to oxidative attack by the laccase and peroxidase system characteristic of white-rot fungi, and fungal enzymes are capable of altering the chemical structure and toxicological properties of pesticides [51]; microbial decomposition is considered one of the primary processes governing the environmental fate of fungicides [52].

### 3.2. Potential for Growth and Tolerance of Pycnoporus sanguineus (NUOL-PPK.02) in Chlorpyrifos- and Azoxystrobin-Contaminated Biomixture

#### 3.2.1. Mycelium Growth and Inhibition on Chlorpyrifos

The effect of increasing chlorpyrifos concentrations on the mycelial growth of *P. sanguineus* was significant. Generally, mycelial growth in uncontaminated biomixture reached 5.00, 6.53, and 8.00 cm at 7, 14, and 30 days, respectively, at 30 ± 2 °C. In the chlorpyrifos-treated biomixture, mycelial growth was progressively reduced with increasing concentration, as shown in Table 2. At 7 days, growth reached 4.50, 4.00, and 2.46 cm at 16.3, 24.5 and 32.7 mg a.i. kg^−1^, respectively; at 14 days, values were 6.06, 5.33, and 3.93 cm; and at 30 days, 7.50, 6.80, and 5.93 cm, respectively. In addition to growth suppression, chlorpyrifos caused a visible reduction in the mycelial growth of *P. sanguineus* (Figure 3a), with inhibition ranging from 6–10% at 16.3 mg a.i. kg^−1^, 15–20% at 24.5 mg a.i. kg^−1^, and 26–51% at 32.7 mg a.i. kg^−1^. Despite this inhibitory effect, *P. sanguineus* was still able to grow in chlorpyrifos-contaminated biomixture, with the extent of growth depending on the concentration applied. Mycelial growth therefore continued, and the degree of inhibition decreased with incubation time. Mycelia growing in chlorpyrifos-contaminated biomixture retained a dense, radiating habit and a white to pale-orange colour throughout the incubation (Figure 4a–c).

It should be emphasised that the strain studied here, *P. sanguineus* NUOL-PPK.02, did not measurably degrade chlorpyrifos: although it tolerated the insecticide and continued to grow in the contaminated biomixture (Table 3), removal in inoculated containers did not differ significantly from that in the non-inoculated controls (Section 3.1.1). This finding is specific to the strain and conditions tested and does not exclude fungal degradation of chlorpyrifos in general. Fungi able to transform chlorpyrifos are known in the literature, including the white-rot fungus *Phanerochaete chrysosporium* [53] and indigenous fungal isolates that removed chlorpyrifos residues under laboratory conditions [42]. Microbial degradation of chlorpyrifos generally begins with hydrolysis of the phosphorothioate ester to 3,5,6-trichloro-2-pyridinol, followed by further transformation of the pyridinol ring [14,43], and bacteria such as *Enterobacter* strain B-14 can use chlorpyrifos as a source of carbon and phosphorus [54]. Screening of additional white-rot strains, or co-inoculation of *P. sanguineus* with microorganisms that hydrolyse chlorpyrifos, would therefore be required before fungal inoculation of chlorpyrifos-contaminated biomixtures could be recommended.

#### 3.2.2. Mycelium Growth and Inhibition on Azoxystrobin

The effect of azoxystrobin on the mycelial growth of *P. sanguineus* was significant across all concentration levels when compared with uncontaminated controls, as shown in Table 2. *P. sanguineus* grew to 4.00, 5.30, and 6.56 cm at 7, 14, and 30 days, respectively, at 10.2 mg a.i. kg^−1^ (the lowest concentration); at 15.3 mg a.i. kg^−1^, growth was 3.03, 4.58, and 5.83 cm; and at 20.4 mg a.i. kg^−1^ (highest concentration), growth was 2.00, 3.63, and 4.96 cm. These values were consistently lower than the uncontaminated control, which reached 5.00, 6.53, and 8.00 cm at 7, 14, and 30 days, respectively, at 30 ± 2 °C.

As a fungicide, azoxystrobin directly inhibited the mycelial growth of *P. sanguineus* in a concentration-dependent manner. At the highest azoxystrobin concentration, mycelia became noticeably thin and attenuated. Overall, mycelial inhibition ranged from 18–20% at 10.2 mg a.i. kg^−1^, 27–39% at 15.3 mg a.i. kg^−1^, and 38–60% at 20.4 mg a.i. kg^−1^ (Figure 3b), with inhibition decreasing over time as the fungus acclimated to the fungicide. Although *P. sanguineus* also tolerated azoxystrobin, mycelial growth was markedly thin and darkened to a deep orange colour at all concentrations, indicating a physiological stress response (Figure 4d–f). Azoxystrobin has been reported to inhibit fungal activity [55], decrease the total phylloplane fungi population [56], and disturb soil fungi structure [57]. However, azoxystrobin did not exert an obvious effect on the fungus population or protease activity [58]. Importantly, fungi can be extremely helpful in removing organic contaminants, including fungicides, and enzymes produced by these organisms can transform the chemical structure of fungicides into less toxic forms [59,60].

### 3.3. Effect of Pycnoporus sanguineus (NUOL-PPK.02) on the Properties of the Biomixture Contaminated with Pesticides (Chlorpyrifos and Azoxystrobin)

The physicochemical properties of the raw materials used as bedding, including soil, sawdust, and rice bran, provided suitable nutritional conditions to support the growth of *P. sanguineus* in pesticide (chlorpyrifos and azoxystrobin)-contaminated biomixture. The soil was collected from an agricultural field with no history of pesticide application; the soil, sawdust and rice bran were all sieved (<0.5 mm) before the experiment was set up. As shown in Table 3, these materials were suitable for the growth of the fungus *P. sanguineus* and for the conduct of the biodegradation experiments.

Contamination of the biomixture with chlorpyrifos and azoxystrobin significantly influenced its physicochemical and nutrient properties following 30 days of incubation with *P. sanguineus.* The physical and chemical characteristics of chlorpyrifos-contaminated biomixture are shown in Table 4, and those of azoxystrobin-contaminated biomixture are shown in Table 5. Biomixture pH, which ranged from 5.7 to 6.7 at the initial stage, increased to 7.6–8.0 after 30 days. Because a comparable rise occurred in the non-inoculated control (from 6.59 to 7.57), the increase is attributable largely to the calcium carbonate added to the biomixture, with fungal activity contributing at most a small additional effect; fungi can nevertheless modify the pH of their substrate through the release of alkaline metabolites and organic acids [61]. Electrical conductivity (EC), exchangeable potassium (Exch. K), organic carbon (OC), the C:N ratio and moisture content for the same treatments are reported in Supplementary Tables S11 and S12. EC, in contrast, decreased in every treatment, from 0.60–0.88 to 0.47–0.58 dS m^−1^, including the non-inoculated control (from 0.60 to 0.51 dS m^−1^); fungi can influence electrical conductivity indirectly through their role in nutrient cycling and organic matter decomposition. The differential effect of fungi on EC under contaminated versus uncontaminated conditions has been previously reported [62]. Cation exchange capacity (CEC) increased by 3–34% in all treatments except the highest application rate of each pesticide (−7.0% for chlorpyrifos and −0.3% for azoxystrobin), reflecting changes in the surface chemistry of the biomixture driven by fungal activity. CEC values provide important insights into nutrient retention dynamics, suggesting a role of fungi in enhancing the nutrient-retention capacity of the matrix. Total nitrogen (TN) changed little in most treatments but increased at the two higher azoxystrobin rates (by 11.7% and 28.2%; Table 5). The nitrogen contained in the applied azoxystrobin (<3 mg N kg^−1^ at the highest rate) is far too small to account for this change, which more plausibly reflects the relative enrichment of nitrogen as organic carbon from the rice bran and sawdust was mineralised during fungal growth. Fungi also assimilate nitrogen for cell wall biosynthesis, and their metabolic activity is known to enhance nitrogen cycling in soil [62,63]. Available phosphorus (Avail. P) increased in every inoculated treatment, by 90.4% in the uncontaminated inoculated biomixture and by progressively smaller amounts as the pesticide rate rose (84.1% to 19.6% for chlorpyrifos and 55.7% to 34.8% for azoxystrobin), whereas it declined slightly in the non-inoculated control (−2.3%); fungal decomposition of the organic components evidently released phosphorus into bioavailable forms, and the pesticides suppressed this effect. Fungi are recognised for their role in phosphorus solubilisation in contaminated soils [64]. The elevated exchangeable potassium (Exch. K) values across treatments are largely attributable to the high potassium content of the bedding materials: soil, sawdust, and rice bran. Exch. K increased most markedly in the uncontaminated inoculated biomixture (by 28.4%, to 841.44 mg kg^−1^) and in the non-inoculated control (by 18.5%), rose slightly at the lowest chlorpyrifos rate (by 2.1%), and declined by 1.1–12.0% in the other contaminated treatments (Supplementary Tables S11 and S12). Exchangeable K nevertheless remained high in all treatments. Fungi play a crucial role in potassium (K+) solubilisation and uptake, thereby influencing soil fertility. Various fungi have been found to enhance potassium uptake in plants under limited K+ conditions [65,66]. Organic matter (OM) content increased most prominently in biomixture inoculated with *P. sanguineus* without pesticide contamination (41.11%), while OM increased by 36.54% at 16.3 mg a.i. kg^−1^ and by 18.09% at 32.7 mg a.i. kg^−1^ chlorpyrifos, reflecting the inhibitory effect of higher pesticide concentrations on fungal biomass production and organic matter transformation. Fungal mycelial activity is well known to influence soil organic matter dynamics, including its accumulation and mineralisation [67]. In chlorpyrifos-contaminated biomixture, the C:N ratio increased by 31–67% at the two lower rates but fell from 33.3 to 13.9 at the highest rate; in azoxystrobin-contaminated biomixture, it rose by 27% at the lowest rate and changed little at the higher rates, remaining between 32.1 and 37.4 after 30 days (Supplementary Tables S11 and S12). These shifts reflect the balance between carbon mineralisation and nitrogen immobilisation associated with the growth of *P. sanguineus* in the biomixture. Biomixture moisture content declined in all treatments during the 30-day incubation, most strongly in the non-inoculated control (from 30.4% to 11.3%) and least in the uncontaminated inoculated biomixture (from 31.0% to 27.2%), with intermediate values (18.8–24.6%) in the contaminated inoculated treatments. In addition, fungi exhibit varying responses to soil moisture levels, impacting their growth and survival [68]. Fungi are known for their biocontrol abilities, especially in wet soils [69]. Collectively, these findings demonstrate that inoculation of the pesticide-contaminated biomixture with *P. sanguineus* (NUOL-PPK.02) is associated with changes in its physicochemical and nutrient properties, most clearly in available phosphorus, CEC and organic matter content, whereas the rise in pH and the fall in EC occurred in the non-inoculated control as well. Because these changes were measured in an autoclaved soil-based biomixture enriched with sawdust and rice bran rather than in a natural soil, they indicate the potential of the approach for biobed-type treatment matrices; whether comparable improvements occur in non-sterile agricultural soils remains to be tested.

Growth of *P. sanguineus* in chlorpyrifos-contaminated biomixture was significantly related to biomixture properties (Figure 5a): it was positively correlated with CEC (r = 0.51, *p* < 0.05) and available phosphorus (r = 0.68, *p* < 0.01) and negatively correlated with exchangeable potassium (r = −0.94, *p* < 0.001). Inhibition of mycelial growth was negatively correlated with the C:N ratio (r = −0.70, *p* < 0.01), and fungal decomposition of chlorpyrifos was positively correlated with its half-life (r = 0.99, *p* < 0.001). In azoxystrobin-contaminated biomixture (Figure 5b), the half-life of azoxystrobin was positively correlated with the inhibition of fungal growth (r = 0.95, *p* < 0.001), fungal decomposition of azoxystrobin was negatively correlated with CEC (r = −0.67, *p* < 0.01), and fungal growth was negatively correlated with exchangeable potassium (r = −0.95, *p* < 0.001).

### 3.4. Nominal Versus Measured Concentrations

Measured day 0 concentrations were 27–51% of nominal and differed by only 1.17- to 1.26-fold across the three application rates. Two explanations were considered. The first is analytical. Matrix-matched calibration covered 0.005 to 0.2 mg L^−1^, well below the concentrations encountered in these samples; extracts were therefore diluted and re-injected until the response fell within that interval, so no reported value was obtained by extrapolation beyond the calibration curve. A ceiling imposed by the top of the curve can accordingly be excluded, and the data are in any case inconsistent with one. Residues continued to fall well below the day 0 values, reaching 5.73 mg kg^−1^ for chlorpyrifos and 1.14 mg kg^−1^ for azoxystrobin by day 60, and the day 0 values themselves remained ordered by application rate; a saturating response would have produced neither.

The second explanation is incomplete recovery from the matrix. The biomixture contained 200 g of sawdust and 200 g of rice bran per container and 8.5% organic matter, and 24 h elapsed between application and the day 0 sampling, which is ample for sorption to proceed. This is consistent with the ratio of measured to nominal concentration falling as the application rate rose (51%, 40% and 32% for chlorpyrifos), and with the sorption behaviour of the two compounds: chlorpyrifos, with a K_oc_ of about 5509 mL g^−1^, would be expected to bind more strongly and to be extracted less completely than azoxystrobin at 589 mL g^−1^ [18,19]. The shortfall is therefore attributed to sorption during the interval between application and sampling rather than to a limitation of the determination.

Because every comparison reported here is between an inoculated and a non-inoculated container of the same application rate, prepared from the same biomixture batch and analysed in the same run against the same calibration, a systematic under-recovery affects both series equally and cancels in the percentage removal. The dissipation results are therefore unaffected by this uncertainty, although the absolute concentrations should be read as conservative.

## 4. Conclusions

Soils in banana-growing regions of Southeast Asia receive an organophosphate insecticide and a strobilurin fungicide of contrasting degradation chemistry, and a remediation agent for these systems must therefore act on more than one class of compound. This study asked whether *Pycnoporus sanguineus* (NUOL-PPK.02), a white-rot fungus isolated in Lao PDR, could meet that requirement in an autoclaved soil-based biomixture under controlled incubation conditions. The answer differed sharply between the two pesticides. Azoxystrobin was removed far more rapidly in the presence of the fungus than without it, and the effect was consistent across all three application rates. Chlorpyrifos, by contrast, dissipated at much the same rate whether the fungus was present or not; the strain tolerated and grew in chlorpyrifos-contaminated biomixture, but its contribution to removal was small and not consistently significant. The contrast is best explained by the strong sorption of chlorpyrifos in an organic-rich matrix and by the greater resistance of its phosphorothioate ester to ligninolytic oxidation. Both pesticides inhibited mycelial extension in proportion to the concentration applied, yet inhibition diminished with time and the fungus continued to colonise the biomixture, indicating a capacity to acclimate rather than merely to survive. Inoculation was also associated with increases in available phosphorus, cation exchange capacity and organic matter content, and this dual action is the practical argument for the approach. Physical and chemical remediation of pesticide-contaminated soil—excavation and replacement, chemical oxidation, and thermal treatment—removes the contaminant at the cost of soil structure, organic matter and biological activity, so that a remediated soil is often an impoverished one. Here, the fungus acted on the contaminant and left the matrix in better chemical condition than it found it, which is the combination a remediation treatment ultimately requires, although this has yet to be demonstrated in natural soil. The improvement should not be attributed to the fungus alone: the biomixture contained sawdust and rice bran, whose decomposition would itself release nitrogen and organic carbon, and the sterilised matrix carried no competing microflora. The increases in available phosphorus and organic matter were nevertheless consistent across both experiments and at every application rate, and this points to fungal inoculation of biobed-type matrices as a treatment that improves the chemical condition of the matrix as it remediates it. These findings position *P. sanguineus* (NUOL-PPK.02) as a promising agent for the treatment of strobilurin-contaminated biomixtures and biobed-type systems rather than as a general-purpose pesticide degrader, and they should be read with the limitations of the design in mind: a sterilised soil-based biomixture rather than a living field soil, a narrow range of measured initial concentrations, no measurement of degradation products, and an identification of the strain based on morphological characters alone, with confirmation by ITS rDNA sequencing still in progress. The absolute residue concentrations should also be read as conservative since the proportion of applied pesticide recoverable from this organic-rich matrix was not established independently, although every value reported lies within the calibrated range of the determination; the comparisons between inoculated and non-inoculated containers, on which every conclusion rests, are not affected. Work now underway addresses these constraints directly through quantification of the chlorpyrifos metabolite 3,5,6-trichloro-2-pyridinol, characterisation of the ligninolytic enzymes expressed under pesticide stress, and trials in non-sterile field soil.

## Figures and Tables

**Figure 1:**
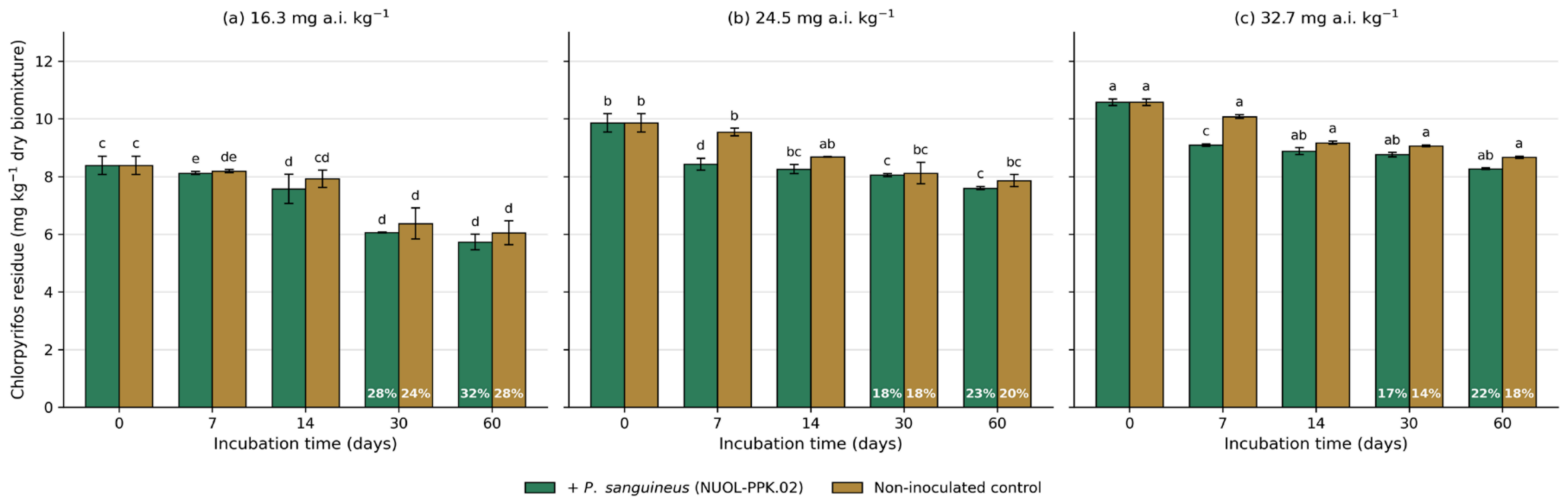
Dissipation of chlorpyrifos (CPS) in contaminated biomixture incubated with and without *Pycnoporus sanguineus* (NUOL-PPK.02) over 0, 7, 14, 30 and 60 days: (**a**) 16.3 mg a.i. kg^−1^, (**b**) 24.5 mg a.i. kg^−1^ and (**c**) 32.7 mg a.i. kg^−1^ nominal application rate. CPS, chlorpyrifos; PPK.02, *P. sanguineus* NUOL-PPK.02. Green bars, inoculated; gold bars, non-inoculated control; percentages within the bars are the reduction from the day 0 concentration. Values are means of three replicate containers; vertical bars denote one standard deviation. Means followed by the same letter within a sampling time do not differ significantly; letters compare all six treatments of the pesticide at that sampling time, i.e., across the three panels (Tukey’s HSD, *p* ≤ 0.05). The *y*-axis reports measured residue concentration in mg kg^−1^ dry biomixture; nominal application rates are given in the panel headings.

**Figure 2:**
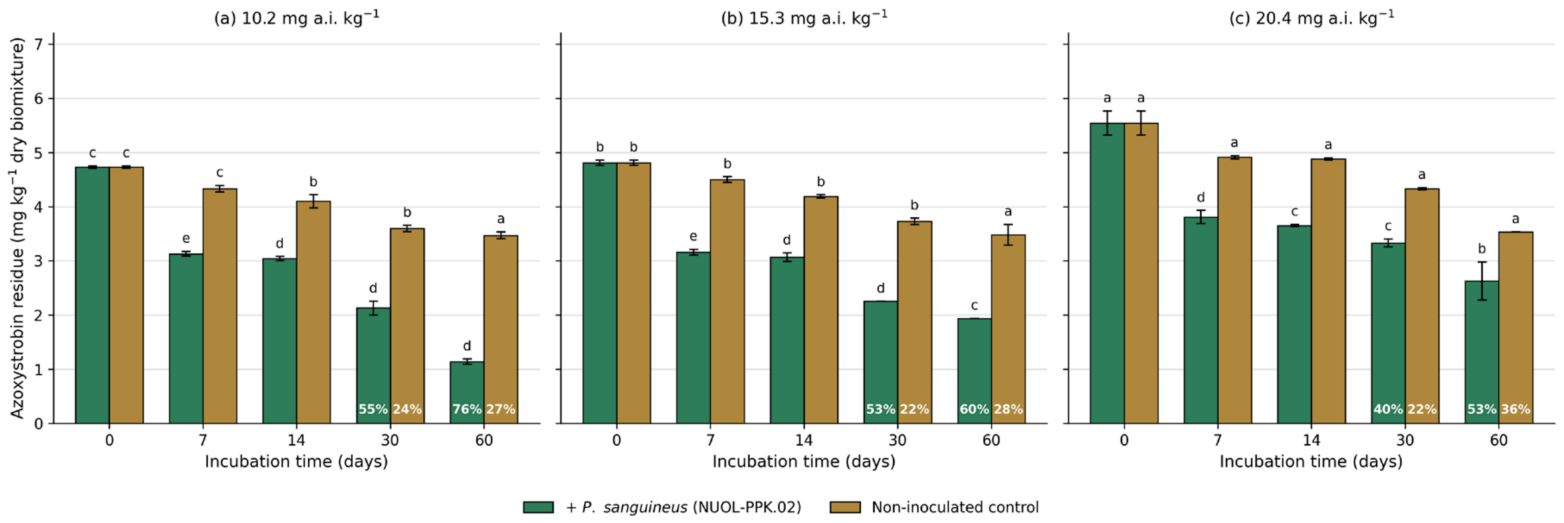
Dissipation of azoxystrobin (AZOXY) in contaminated biomixture incubated with and without *Pycnoporus sanguineus* (NUOL-PPK.02) over 0, 7, 14, 30 and 60 days: (**a**) 10.2 mg a.i. kg^−1^, (**b**) 15.3 mg a.i. kg^−1^ and (**c**) 20.4 mg a.i. kg^−1^ nominal application rate. AZOXY, azoxystrobin; PPK.02, *P. sanguineus* NUOL-PPK.02. Green bars, inoculated; gold bars, non-inoculated control; percentages within the bars are the reduction from the day 0 concentration. Values are means of three replicate containers; vertical bars denote one standard deviation. Means followed by the same letter within a sampling time do not differ significantly; letters compare all six treatments of the pesticide at that sampling time, i.e., across the three panels (Tukey’s HSD, *p* ≤ 0.05). Measured initial concentrations differed by only 1.17-fold across the three application rates (see Section 3.1.2 and Supplementary Table S3).

**Figure 3:**
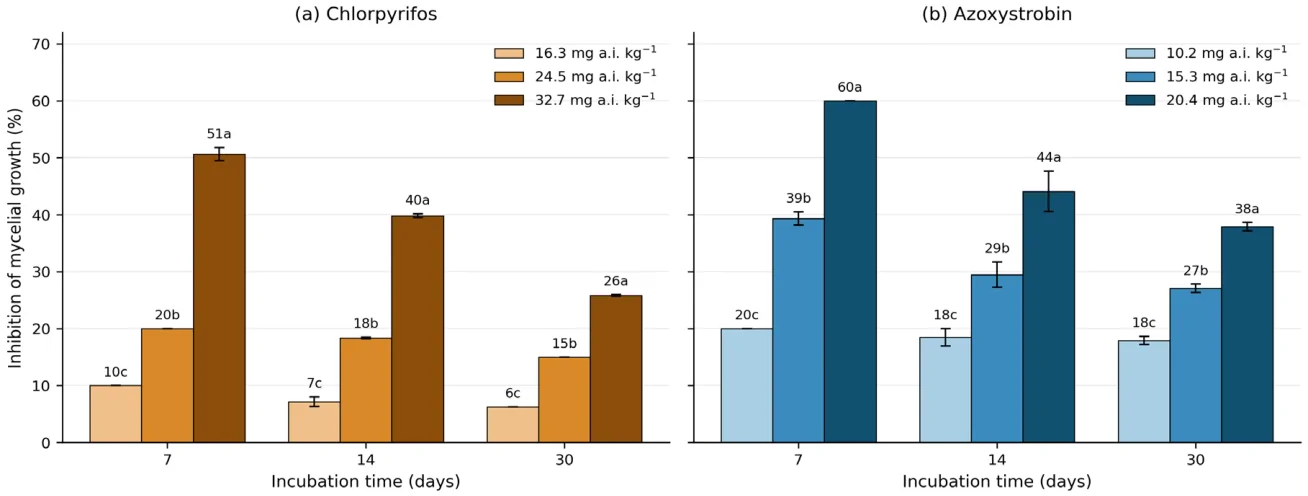
Inhibition of mycelial growth of *Pycnoporus sanguineus* (NUOL-PPK.02) in (**a**) chlorpyrifos- and (**b**) azoxystrobin-contaminated biomixture at 7, 14 and 30 days of incubation, expressed as percentage inhibition relative to the uncontaminated inoculated control. Values are calculated from the mean mycelial extension of three replicate containers per treatment; the calculation is given in Section 2.4. Shading within each panel denotes the nominal application rate; vertical bars denote one standard deviation, and letters above the bars indicate mean separation among application rates within a sampling time (Tukey’s HSD, *p* ≤ 0.05).

**Figure 4:**
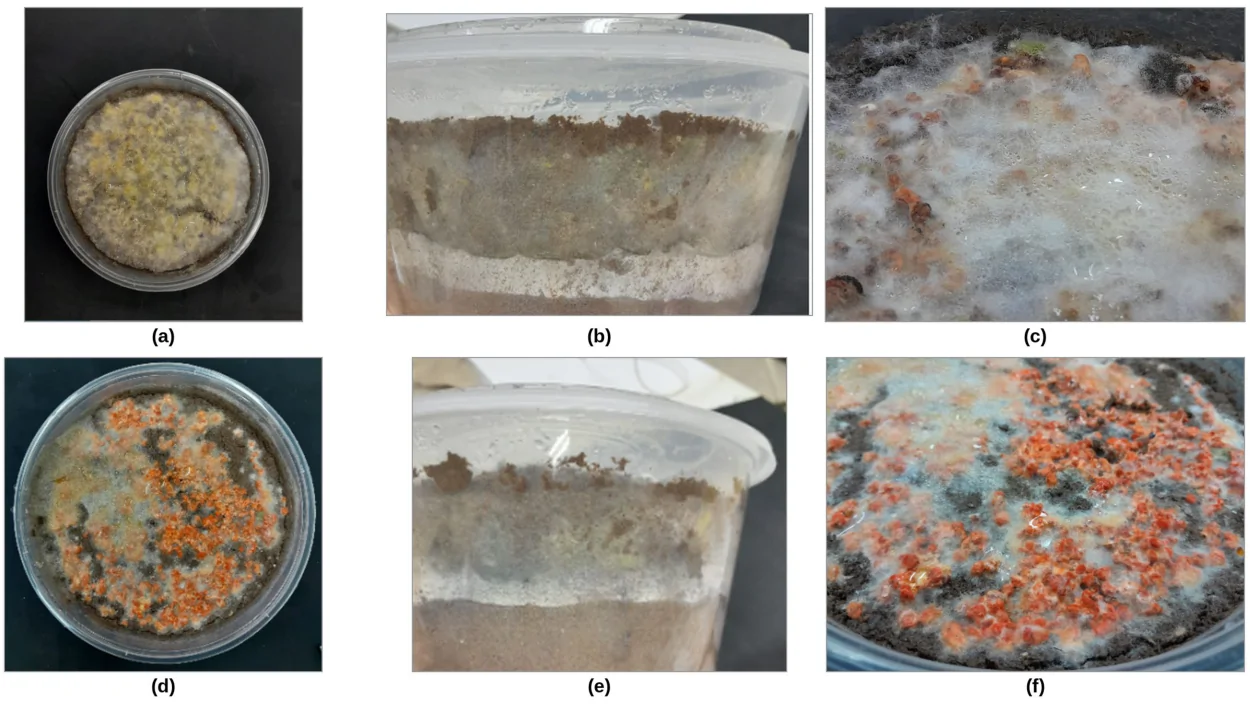
Mycelial characteristics of *Pycnoporus sanguineus* (NUOL-PPK.02) under pot conditions. (**a**–**c**) morphology and hyphal character growing on lowest rate of chlorpyrifos; (**d**–**f**) on lowest rate of azoxystrobin.

**Figure 5:**
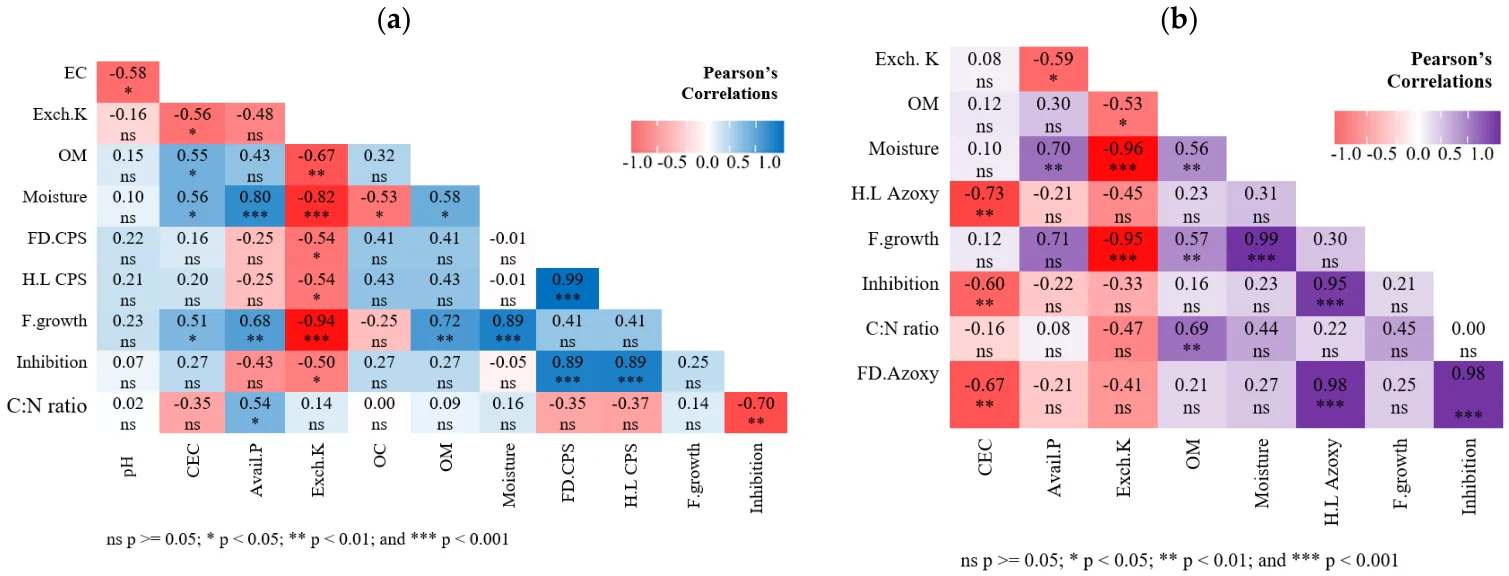
Correlation potential of *Pycnoporus sanguineus* (NUOL-PPK.02) in the biodegradation of contaminated biomixture and its effect on biomixture properties, based on Pearson’s correlation coefficient: (**a**) chlorpyrifos-contaminated biomixture, and (**b**) azoxystrobin-contaminated biomixture. EC, electrical conductivity; OM, organic matter; OC, organic carbon; FD.CPS, fungi decomposing of chlorpyrifos; FD.Azoxy, fungi decomposing of azoxystrobin; H.L CPS, half-life of chlorpyrifos; H.L Azoxy, half-life of azoxystrobin; F. growth, fungal growth; Inhibition, inhibition of mycelial growth; CEC, cation exchange capacity; Exch. K, exchangeable potassium; Avail. P, available phosphorus.

**Table 1:** Physical and chemical characteristics of the raw materials used to prepare the biomixture: soil, sawdust and rice bran (mean ± standard deviation, *n* = 3). These values describe the components before mixing, sterilisation and pesticide application.

Parameters	Soil	Sawdust	Rice Bran
pH (1:2)	5.87 ± 0.23	5.84 ± 0.14	7.28 ± 0.40
EC (dS m^−1^)	0.099 ± 0.005	0.167 ± 0.003	0.002 ± 0.001
CEC (meq 100 g^−1^)	4.86 ± 0.74	33.16 ± 2.68	54.46 ± 5.54
Total Nitrogen (%)	0.10 ± 0.03	0.11 ± 0.01	2.15 ± 0.15
Available Phosphorus (mg kg^−1^)	0.06 ± 0.01	0.24 ± 0.06	2.42 ± 0.56
Exchangeable Potassium (mg kg^−1^)	129.76 ± 4.67	87.01 ± 1.31	335.01 ± 7.07
Organic matter (%)	0.91 ± 0.07	66.37 ± 1.00	49.92 ± 0.01
Organic carbon (%)	0.53 ± 0.07	38.50 ± 0.50	28.96 ± 0.02
C/N ratio	5.43 ± 0.05	346.46 ± 0.25	13.45 ± 0.08
Heavy metal/Cu (mg kg^−1^)	9.68 ± 0.70	2.44 ± 0.39	1.10 ± 0.20

**Table 2:** Treatment structure of the pot incubation experiment with *Pycnoporus sanguineus* (NUOL-PPK.02) in chlorpyrifos- and azoxystrobin-contaminated biomixture. All treatments comprised three replicate containers. T1 and T2 were a single set of control containers common to both pesticides. Application rates are expressed as mg of active ingredient per kg of dry biomixture; the derivation from the volume of formulated product applied is given in Supplementary Table S1.

Treatment	*P. sanguineus* (NUOL-PPK.02) (g)	Pesticide Applied Chlorpyrifos (mg a.i. kg^−1^ Dry Biomixture)	Pesticide Applied Azoxystrobin (mg a.i. kg^−1^ Dry Biomixture)	Contamination Level
T1	0	—	—	Uncontaminated control
T2	25	—	—	Uncontaminated control, fungus only
T3	25	16.3	—	Lowest, inoculated
T4	0	16.3	—	Lowest, non-inoculated (abiotic control)
T5	25	24.5	—	Moderate, inoculated
T6	0	24.5	—	Moderate, non-inoculated (abiotic control)
T7	25	32.7	—	Highest, inoculated
T8	0	32.7	—	Highest, non-inoculated (abiotic control)
T9	25	—	10.2	Lowest, inoculated
T10	0	—	10.2	Lowest, non-inoculated (abiotic control)
T11	25	—	15.3	Moderate, inoculated
T12	0	—	15.3	Moderate, non-inoculated (abiotic control)
T13	25	—	20.4	Highest, inoculated
T14	0	—	20.4	Highest, non-inoculated (abiotic control)

**Table 3:** Mycelial extension (cm) of *Pycnoporus sanguineus* (NUOL-PPK.02) in chlorpyrifos- and azoxystrobin-contaminated biomixture at 7, 14 and 30 days of incubation (mean ± standard deviation, *n* = 3). Application rates are expressed as mg of active ingredient per kg of dry biomixture.

Concentration	Growth of *Pycnoporus sanguineus* (NUOL-PPK.02)		
	7 Days	14 Days	30 Days
Biomixture + PPK.02 (uncontaminated biomixture)	5.00 ± 0.00 a	6.53 ± 0.06 a	8.00 ± 0.00 a
Chlorpyrifos			
Biomixture + 16.3 mg a.i. kg^−1^ of CPS + PPK.02	4.50 ± 0.00 b	6.06 ± 0.06 b	7.50 ± 0.00 b
Biomixture + 24.5 mg a.i. kg^−1^ of CPS + PPK.02	4.00 ± 0.00 c	5.33 ± 0.06 c	6.80 ± 0.00 c
Biomixture + 32.7 mg a.i. kg^−1^ of CPS + PPK.02	2.46 ± 0.06 d	3.93 ± 0.06 d	5.93 ± 0.12 d
F-Test	***	***	***
Azoxystrobin			
Biomixture + 10.2 mg a.i. kg^−1^ of AZOXY + PPK.02	4.00 ± 0.00 b	5.30 ± 0.10 b	6.56 ± 0.06 b
Biomixture + 15.3 mg a.i. kg^−1^ of AZOXY + PPK.02	3.03 ± 0.06 c	4.58 ± 0.14 c	5.83 ± 0.06 c
Biomixture + 20.4 mg a.i. kg^−1^ of AZOXY + PPK.02	2.00 ± 0.00 d	3.63 ± 0.23 d	4.96 ± 0.06 d
F-Test	***	***	***

CPS = Chlorpyrifos; AZOXY = Azoxystrobin; PPK.02 = *Pycnoporus sanguineus* (NUOL-PPK.02); *** = significantly different at *p* ≤ 0.001. A similar letter within the same column indicates no significant difference at *p* = 0.05 between treatments by Tukey’s HSD test.

**Table 4:** The physical and chemical characteristics of chlorpyrifos-contaminated biomixture incubated with *Pycnoporus sanguineus* (NUOL-PPK.02) after 30 days (Mean ± standard deviation). Electrical conductivity, exchangeable potassium, organic carbon, C:N ratio and moisture content for the same treatments are given in Supplementary Tables S11 and S12.

Concentration	Period	Nutrient Content		Organic Content	pH	CEC (meq 100 g^−1^)
		Total N (%)	Avail. P (mg kg^−1^)	OM (%)		
Biomixture control (non-inoculated, uncontaminated)	Initial	0.17 ± 0.02 ab	5.24 ± 0.54 a	8.51 ± 0.65	6.59 ± 0.05	5.21 ± 0.12 bc
	30 days	0.18 ± 0.04	5.12 ± 0.49 c	10.28 ± 0.22	7.57 ± 0.18	6.15 ± 0.12
	%change	6.52	−2.29	20.80	14.87	18.18
Biomixture + PPK.02 (uncontaminated)	Initial	0.17 ± 0.02 ab	4.50 ± 0.41 abc	7.76 ± 0.62	6.60 ± 0.60	5.12 ± 0.70 c
	30 days	0.17 ± 0.01	8.57 ± 1.35 a	10.95 ± 0.17	7.65 ± 0.72	6.86 ± 0.33
	%change	1.37	90.44	41.11	15.91	33.90
Biomixture + 16.3 mg a.i. kg^−1^ of CPS + PPK.02	Initial	0.20 ± 0.0 a	4.08 ± 0.09 c	8.10 ± 0.95	6.56 ± 0.16	5.45 ± 0.25 bc
	30 days	0.16 ± 0.00	7.51 ± 0.36 ab	11.06 ± 0.48	7.97 ± 0.41	6.64 ± 0.56
	%change	−18.52	84.07	36.54	14.87	21.95
Biomixture + 24.5 mg a.i. kg^−1^ of CPS + PPK.02	Initial	0.18 ± 0.00 ab	4.97 ± 0.11 ab	8.89 ± 0.35	6.27 ± 0.18	6.11 ± 0.03 b
	30 days	0.18 ± 0.02	6.47 ± 0.08 bc	10.95 ± 0.44	7.78 ± 0.36	6.30 ± 0.22
	%change	−0.09	30.18	23.17	15.91	3.18
Biomixture + 32.7 mg a.i. kg^−1^ of CPS + PPK.02	Initial	0.15 ± 0.02 b	4.38 ± 0.09 bc	9.23 ± 0.63	5.70 ± 1.21	7.44 ± 0.11 a
	30 days	0.16 ± 0.25	5.24 ± 0.01 c	10.90 ± 0.01	7.75 ± 0.57	6.92 ± 0.05
	%change	−0.69	19.63	18.09	21.49	−6.99

CPS = Chlorpyrifos, NUOL-PPK.02 = *Pycnoporus sanguineus* (NUOL-PPK.02); similar letter within the same column indicates no significant difference at *p* = 0.05 between treatments by Tukey’s HSD test.

**Table 5:** The physical and chemical characteristics of azoxystrobin-contaminated biomixture incubated with *Pycnoporus sanguineus* (NUOL-PPK.02) after 30 days (Mean ± standard deviation). Electrical conductivity, exchangeable potassium, organic carbon, C:N ratio and moisture content for the same treatments are given in Supplementary Tables S11 and S12.

Concentration	Period	Nutrient Content		Organic Content	pH	CEC (meq 100 g^−1^)
		Total N (%)	Avail. P (mg kg^−1^)	OM (%)		
Biomixture control (non-inoculated, uncontaminated)	Initial	0.17 ± 0.02	5.24 ± 0.54	8.51 ± 0.65	6.59 ± 0.05	5.21 ± 0.12 ab
	30 days	0.18 ± 0.04	5.12 ± 0.49 b	10.28 ± 0.22	7.57 ± 0.18	6.15 ± 0.12 ab
	%change	6.52	−2.29	20.80	14.87	18.18
Biomixture + PPK.02 (uncontaminated)	Initial	0.17 ± 0.02	4.50 ± 0.41 abc	7.76 ± 0.62	6.60 ± 0.60	5.12 ± 0.70 ab
	30 days	0.17 ± 0.01	8.57 ± 1.35 a	10.95 ± 0.17	7.65 ± 0.72	6.86 ± 0.33 a
	%change	1.37	90.44	41.11	15.91	33.90
Biomixture + 10.2 mg a.i. kg^−1^ of AZOXY + PPK.02	Initial	0.16 ± 0.01	4.20 ± 0.55	8.42 ± 0.74	6.68 ± 0.09	4.46 ± 0.03 b
	30 days	0.15 ± 0.00	6.54 ± 0.46 ab	10.89 ± 0.52	7.94 ± 0.12	5.52 ± 0.08 b
	%change	1.69	55.71	29.33	18.86	23.77
Biomixture + 15.3 mg a.i. kg^−1^ of AZOXY + PPK.02	Initial	0.15 ± 0.02	4.51 ± 0.41	8.63 ± 0.12	6.65 ± 0.01	4.98 ± 0.45 ab
	30 days	0.17 ± 0.01	6.47 ± 0.55 b	10.89 ± 0.73	7.87 ± 0.08	5.75 ± 0.41 b
	%change	11.67	43.46	26.19	18.35	15.46
Biomixture + 20.4 mg a.i. kg^−1^ of AZOXY + PPK.02	Initial	0.15 ± 0.00	4.57 ± 0.74	8.67 ± 0.40	6.51 ± 0.12	5.88 ± 0.36 a
	30 days	0.19 ± 0.00	6.16 ± 1.10 b	10.17 ± 0.20	7.58 ± 0.07	5.86 ± 0.27 b
	%change	28.19	34.79	17.30	16.44	−0.34

AZOXY = Azoxystrobin; PPK.02 = *Pycnoporus sanguineus* (NUOL-PPK.02); similar letter within the same column indicates no significant difference at *p* = 0.05 between treatments by Tukey’s HSD test.

## Data Availability

The original contributions presented in this study are included in the article/Supplementary Materials. Further inquiries can be directed to the corresponding author.

## Supplementary Materials

The following supporting information can be downloaded at https://www.mdpi.com/article/10.3390/toxics14100839/s1, Table S1: Derivation of the nominal pesticide application rates; Table S2: Liquid chromatography gradient programme; Table S3: Nominal application rates and measured initial concentrations; Table S4: Precursor and product ions used for quantification and confirmation; Table S5: Method performance for the determination of chlorpyrifos and azoxystrobin; Table S6: Experimental set-up (Table 1 of the article); Table S7: Physicochemical characteristics of the raw materials (Table 3); Table S8: Chlorpyrifos residues in the biomixture (Figure 1); Table S9: Azoxystrobin residues in the biomixture (Figure 2); Table S10: Mycelial growth and percentage inhibition (Table 2 and Figure 3); Table S11: Biomixture physicochemical properties, chlorpyrifos experiment. This table gives the full parameter set, including electrical conductivity, exchangeable potassium, organic carbon, C:N ratio and moisture content (at the start of incubation and after 30 days), which are not shown in Table 4 of the article; Table S12: Biomixture physicochemical properties, azoxystrobin experiment. This table gives the full parameter set, including electrical conductivity, exchangeable potassium, organic carbon, C:N ratio and moisture content (at the start of incubation and after 30 days), which are not shown in Table 5 of the article; Figure S1: Measurement of mycelial extension in (a) chlorpyrifos- and (b) azoxystrobin-contaminated biomixture under container conditions. Extension was measured as the vertical distance from the biomixture surface, taken as the reference point, to the lowest visible point of mycelial growth against the container wall, recorded with a ruler at 7, 14 and 30 days. The marks in panel (b) indicate the position of the mycelial front at day 0 and at day 7. One measurement was made per container at each sampling time, and the value reported for a treatment is the mean of its three replicate containers.
