# Supplementary material for: Potential of Pycnoporus sanguineus (NUOL-PPK.02) for Bioremediation of Chlorpyrifos and Azoxystrobin in an Autoclaved Soil-Based Biomixture: A Pot Incubation Study
Source: Toxics. 2026 Sep 22;14(10):839. doi: 10.3390/toxics14100839 (PMC13709529; doi:10.3390/toxics14100839)
Supplement: Supplementary file 1 [file toxics-14-00839-s001.zip › toxics-4533192-supplementary.pdf]

Supplementary Material

Potential of *Pycnoporus sanguineus* (NUOL-PPK.02) for Bioremediation of Chlorpyrifos and Azoxystrobin in an Autoclaved Soil-Based Biomixture: A Pot Incubation Study

Nuttaya Xasydavong, Phoutthasone Sibounnavong, Noppol Arunrat, Jarupong Prasopsuk and Chuleemas Boonthai Iwai*

Table S1. Derivation of the nominal pesticide application rates

Pesticide	Product applied (µL)	a.i. applied (mg)	Applied solution (mg L^{-1})	Nominal rate (mg a.i. kg^{-1} DW)
Chlorpyrifos	100	40.0	200.0	16.33
Chlorpyrifos	150	60.0	300.0	24.49
Chlorpyrifos	200	80.0	400.0	32.65
Azoxystrobin	100	25.0	125.0	10.20
Azoxystrobin	150	37.5	187.5	15.31
Azoxystrobin	200	50.0	250.0	20.41

Formulations: chlorpyrifos 40% w/v EC (TIGER; 400 g a.i. L^{-1} = 400 mg a.i. mL^{-1}) and azoxystrobin 25% w/v SC (MISTA; 250 g a.i. L^{-1} = 250 mg a.i. mL^{-1}). Each dose of formulated product was pipetted into 200 mL of distilled water and applied evenly to 2.450 kg (dry weight) of biomixture in one container, which was then mixed thoroughly. Worked example (chlorpyrifos, 100 µL): mass of a.i. = 0.100 mL × 400 mg mL^{-1} = 40.0 mg; applied solution = 40.0 mg ÷ 0.200 L = 200.0 mg L^{-1}; nominal rate = 40.0 mg ÷ 2.450 kg = 16.33 mg a.i. kg^{-1} DW. DW = dry weight of biomixture.

Table S2. Liquid chromatography gradient programme

Time (min)	Flow (mL min^{-1})	Mobile phase A (%)	Mobile phase B (%)
0.00	0.20	99.0	1.0
1.00	0.20	99.0	1.0
3.00	0.20	61.0	39.0
14.00	0.40	0.1	99.9
16.00	0.48	0.1	99.9
16.10	0.48	99.0	1.0
19.00	0.48	99.0	1.0
19.10	0.20	99.0	1.0
20.00	0.20	99.0	1.0

Mobile phase A: 10% methanol; mobile phase B: methanol; both containing 5 mM ammonium formate and 0.01% formic acid. Column: Thermo Scientific Acclaim RSLC 120 C18 (2.1 × 100 mm, 2.2 µm, 120 Å) held at 30 °C; injection volume 5 µL; total cycle time 20 min.

Table S3. Nominal application rates and measured initial concentrations

Pesticide	Product (µL)	Nominal (mg kg^{-1})	Measured day 0 (mg kg^{-1})	Measured / nominal (%)	Nominal ratio	Measured ratio
Chlorpyrifos	100	16.33	8.39 ± 0.32	51.4	1.00	1.00
Chlorpyrifos	150	24.49	9.86 ± 0.32	40.3	1.50	1.18
Chlorpyrifos	200	32.65	10.58 ± 0.11	32.4	2.00	1.26
Azoxystrobin	100	10.20	4.73 ± 0.02	46.4	1.00	1.00
Azoxystrobin	150	15.31	4.81 ± 0.05	31.4	1.50	1.02
Azoxystrobin	200	20.41	5.54 ± 0.22	27.1	2.00	1.17

Measured values are the concentrations determined 24 h after pesticide application (day 0), expressed as means ± standard deviation of three replicate containers on a dry-weight basis. Residues were determined by the QuEChERS procedure of BS EN 15662:2018 (Table S3–S5). Ratios express each rate relative to the lowest rate of the same pesticide. Because the measured initial concentrations differed by only 1.26-fold (chlorpyrifos) and 1.17-fold (azoxystrobin) across the three application rates, all dissipation parameters were calculated from measured rather than nominal concentrations, and the results are not interpreted in dose–response terms.

Table S4. Precursor and product ions used for quantification and confirmation

Compound	Precursor ion (m/z)	Quantifier ion (m/z)	Qualifier ion (m/z)
Azoxystrobin	404.1241	372.0979	344.1030
Chlorpyrifos	349.9336	197.9275	114.9614

Precursor ions are the protonated molecules $[M+H]^+$. Spectra were acquired on a Bruker Compact ESI-Q-TOF instrument in broadband collision-induced dissociation (bbCID) mode. Because a Q-TOF acquires full high-resolution spectra rather than discrete selected-reaction transitions, quantification was based on extracted ion chromatograms of the quantifier ion, with identity confirmed by the qualifier ion and by mass accuracy within ± 5 ppm.

Table S5. Method performance for the determination of chlorpyrifos and azoxystrobin

Compound	r^2	LOD (mg kg^{-1})	LOQ (mg kg^{-1})	Recovery (%)	RSD (%)
Azoxystrobin	0.9957	0.001	0.01	90.9	5.83
Chlorpyrifos	0.9961	0.001	0.01	114.2	5.02

Determined with matrix-matched calibration standards prepared in blank biomixture extract at five concentrations (0.005, 0.01, 0.05, 0.1 and 0.2 mg L^{-1}). Extraction used 10 g of biomixture in 10 mL of acetonitrile, so that 1 mg L^{-1} in the extract corresponds to 1 mg kg^{-1} in the biomixture; extracts whose response exceeded the highest calibration point were diluted and re-injected, so every reported value lies within the calibrated interval. Recovery was assessed by spiking blank biomixture at 0.1 mg kg^{-1} in triplicate. Acceptance criteria: recovery 70–120%, RSD < 20%. Analyses were carried out at the Office of Agricultural Research and Development Region 3, Department of Agriculture, Khon Kaen, Thailand.

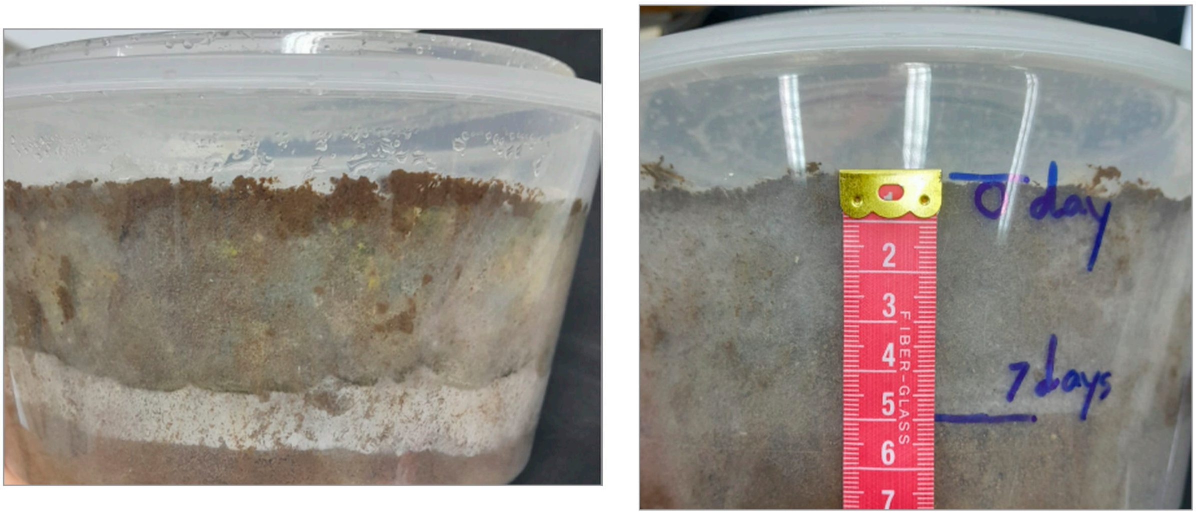

(a) (b)

Figure S1. Measurement of mycelial extension in (a) chlorpyrifos- and (b) azoxystrobin-contaminated biomixture under container conditions. Extension was measured as the vertical distance from the biomixture surface, taken as the reference point, to the lowest visible point of mycelial growth against the container wall, recorded with a ruler at 7, 14 and 30 days. The marks in panel (b) indicate the position of the mycelial front at day 0 and at day 7. One measurement was made per container at each sampling time, and the value reported for a treatment is the mean of its three replicate containers.

Supplementary dataset

The complete underlying dataset is supplied as a separate spreadsheet, Supplementary Dataset toxics-4533192.xlsx, which contains the following tables. Each sheet gives the treatment, the nominal application rate (mg a.i. kg^{-1} dry biomixture), the sampling time, and the mean, standard deviation, number of replicates, coefficient of variation and Tukey grouping for every measurement.

Table S6. Experimental set-up (Table 1 of the article).

Table S7. Physicochemical characteristics of the raw materials (Table 3).

Table S8. Chlorpyrifos residues in the biomixture (Figure 1).

Table S9. Azoxystrobin residues in the biomixture (Figure 2).

Table S10. Mycelial growth and percentage inhibition (Table 2 and Figure 3).

Table S11. Biomixture physicochemical properties, chlorpyrifos experiment. This table gives the full parameter set, including electrical conductivity, exchangeable potassium, organic carbon, C:N ratio and moisture content (at the start of incubation and after 30 days), which are not shown in Table 4 of the article.

Table S12. Biomixture physicochemical properties, azoxystrobin experiment. This table gives the full parameter set, including electrical conductivity, exchangeable potassium, organic carbon, C:N ratio and moisture content (at the start of incubation and after 30 days), which are not shown in Table 5 of the article.
